# FABP5 drives ferroptosis in psoriasis

**DOI:** 10.1038/s41418-025-01629-x

**Published:** 2025-11-29

**Authors:** Zhihao Xu, Li Zhuang, Boyi Gan

**Affiliations:** 1 Department of Experimental Radiation Oncology, The University of Texas MD Anderson Cancer Center, Houston, TX USA; 2 The University of Texas MD Anderson UTHealth Graduate School of Biomedical Sciences, Houston, TX USA

**Keywords:** Cell death and immune response, Cell biology

**Fig. 1:**
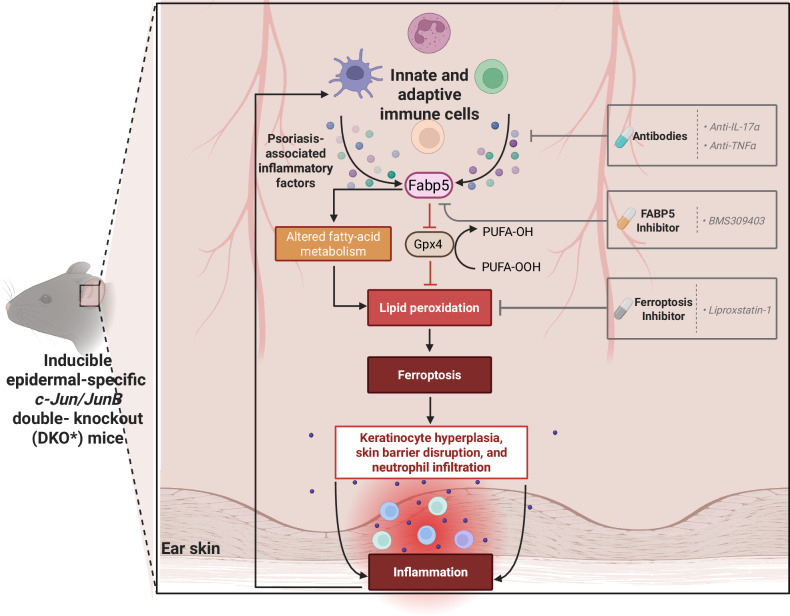
Schematic showing psoriasis-like skin inflammation amplified by the FABP5–GPX4 axis and ferroptosis. In the inducible, epidermal-specific *c-Jun/JunB* double-knockout (DKO*) mice, psoriasiform epidermis exposed to psoriasis-associated inflammatory cytokines (such as IL-17 and TNF-α) exhibits increased FABP5 expression. FABP5 reprograms fatty-acid metabolism (particularly by promoting the incorporation of polyunsaturated fatty acids into membrane phospholipids) and reduces GPX4 expression, thereby weakening GPX4-dependent detoxification of lipid peroxides. Together, these effects trigger ferroptotic stress, leading to keratinocyte hyperplasia, skin barrier disruption, and neutrophil infiltration. The resulting epithelial damage further stimulates immune activation, establishing a self-perpetuating cycle that amplifies psoriatic skin inflammation. Treatment with anti-IL-17 or anti-TNF-α antibody, as well as with the FABP inhibitor BMS-309403 or the ferroptosis inhibitor liproxstatin-1, alleviates psoriatic skin inflammation.

Psoriasis, a chronic inflammatory disorder of the skin and joints, has long been viewed as a prototype of immune-mediated disease [1]. The success of biologics targeting TNF-α, IL-17, and IL-23 has transformed therapies for psoriasis treatment; however, around one-third of patients remain partial responders or relapse over time [2]. This clinical challenge has stimulated a search for additional mechanisms outside canonical cytokine signaling. In a compelling study now published in *Cell Death & Differentiation*, Mieczkowski et al. identify an unexpected metabolic underlying mechanism for this disease: the lipid chaperone fatty-acid-binding protein 5 (FABP5) aggravates psoriatic inflammation by driving keratinocytes into ferroptosis, a form of regulated cell death induced by iron-dependent lipid peroxidation (Fig. 1) [3]. Their findings reposition psoriasis not merely as an immune disorder, but as a disease of dysregulated lipid redox homeostasis.

Keratinocytes, the main constituents of the epidermis, are increasingly recognized as active participants in cutaneous inflammation. They sense stress, release cytokines, and amplify immune responses. However, how metabolic stress within keratinocytes regulates inflammation has remained incompletely understood. Using an inducible, epidermal-specific *c-Jun/JunB* double-knockout (DKO*) mouse model that faithfully recapitulates psoriatic skin and arthritis, the authors reveal profound metabolic alterations characterized by reductions in dermal fat and circulating free fatty acids, as well as elevated β-hydroxybutyrate [3]. Multi-omic analyses of DKO* skin and human psoriatic lesions identify two opposing molecular changes: upregulation of FABP5 and downregulation of the key ferroptosis suppressive protein glutathione peroxidase 4 (GPX4). These shifts — one enhancing lipid transport, the other limiting the detoxification of lipid peroxidation — presumably create cellular conditions that favor oxidative damage and ferroptosis. Correspondingly, DKO* epidermis exhibits increased 4-hydroxynonenal (4-HNE) staining, a biomarker of lipid peroxidation, consistent with ferroptosis induction in this context.

FABP5 normally transports long-chain fatty acids, such as arachidonic acid (a type of polyunsaturated fatty acids [PUFAs]), to intracellular destinations or target proteins including nuclear peroxisome-proliferator-activated receptors (PPARs) [4]. FABP5 also channels PUFAs into cellular membranes, forming PUFA-containing phospholipids that are intrinsically vulnerable to lipid peroxidation and ferroptosis due to their bis-allylic structure with multiple double bonds [5]; consistently, previous studies have shown that FABP5 promotes ferroptosis sensitivity in cancer and neuronal models [6]. The new study extends this concept to skin inflammation and proposes that increased FABP5 expression funnels these redox-active lipids toward cellular membranes while suppressing the expression of GPX4, the lipid peroxidation–detoxifying enzyme and key ferroptosis suppressor, thereby amplifying lipid peroxidation and promoting inflammatory cell recruitment (Fig. 1) [3].

Pharmacological inhibition of FABP activity with BMS-309403 substantiates the causal link between FABP5 and GPX4 [3]. Oral BMS treatment of animals markedly attenuates epidermal thickening, keratinocyte hyperproliferation, and neutrophil infiltration in DKO* mice, while restoring GPX4 and reducing 4-HNE accumulation. Notably, systemic IL-17A and TNF-α levels remain unchanged, indicating that FABP5 most likely acts downstream of cytokine signaling and primarily within the epidermis (Fig. 1). Parallel experiments using the ferroptosis inhibitor liproxstatin-1 show similar protective effects, suggesting that ferroptosis plays a causal role in psoriatic inflammation.

If FABP5 acts downstream of IL-17 and TNF-α, could its modulation feed back to regulate immune pathways? Treatment of DKO* mice with anti-IL-17A or anti-TNF-α antibodies normalized Fabp5 and Gpx4 expression and suppressed lipid peroxidation. Transcriptomic data from psoriasis patients treated with secukinumab or other biologics reveal similar patterns, with decreased FABP5 expression and GPX4 restoration [3]. This crosstalk implies that cytokine-driven upregulation of FABP5 amplifies oxidative stress, which in turn fuels cytokine release from dying keratinocytes (Fig. 1).

How might FABP5 suppress GPX4 expression? FABP5 may interact with PPARβ/δ or other antioxidant transcriptional regulators such as NRF2 to modulate transcriptional networks, thereby indirectly repressing *GPX4* expression [7, 8]. Interestingly, the same research group recently identified an anti-inflammatory role for p62, a modulator of the KEAP1–NRF2 axis, in the same DKO* model [9], suggesting a broader oxidative-stress circuitry governing psoriatic inflammation.

Although ferroptosis was first described in cancer cells [10] and its role in cancer biology is well established [11], its relevance has been extended to other diseases such as neurodegeneration, ischemia, and immune disorders [5]. In the skin, *GPX4* deficiency in basal keratinocytes alone can trigger psoriasiform lesions [12]. The present study fills the missing mechanistic gap by identifying FABP5 as an upstream driver of this process. It reveals psoriasis as a disorder where lipid peroxidation is not a by-product of inflammation but a driver of it. This insight also provides a bridge to comorbid metabolic syndromes frequently seen in psoriasis patients, who display systemic oxidative stress and dyslipidemia [13]. Furthermore, elevated circulating FABP5, which is observed both in patients and DKO* mice, may thus serve as a biomarker connecting cutaneous and metabolic disease.

Regarding the therapeutic implication of this study, FABP inhibitors such as BMS-309403 or ferroptosis inhibitors like liproxstatin-1 might complement existing therapeutic approaches by targeting keratinocyte metabolism. These agents ameliorate skin lesions without altering joint inflammation, consistent with clinical experience that anti-IL-17 or anti-TNF therapies often yield better cutaneous than articular responses. Beyond psoriasis, FABP5 inhibition could be explored in other ferroptosis-linked epithelial disorders such as atopic dermatitis or radiation-induced dermatitis, where oxidative lipid damage undermines barrier integrity. However, given FABP5’s broad metabolic roles, tissue-restricted or transient inhibition will likely be needed to avoid systemic lipid perturbation. Nonetheless, targeting the FABP5–GPX4 axis could represent a novel adjunct strategy to potentiate current biologics, reduce required dosages, and mitigate side effects.

Taken together, Mieczkowski et al. show that a lipid-binding protein can promote psoriatic inflammation through ferroptosis, thereby linking cellular metabolism to immune signaling [3]. Their work deepens our understanding of psoriasis pathogenesis and points to the therapeutic potential of targeting ferroptosis within the skin’s metabolic machinery. Future studies should clarify how FABP5 regulates GPX4 expression, identify lipid species that initiate keratinocyte ferroptosis, and examine whether circulating FABP5 or oxidized lipid adducts predict therapeutic response. Combining ferroptosis inhibitors with cytokine-targeted therapies could enhance efficacy while reducing immune suppression. Ultimately, targeting epidermal lipid peroxidation may open new paths for treating psoriasis and related chronic inflammatory diseases.
